# Unscented Particle Filter Algorithm Based on Divide-and-Conquer Sampling for Target Tracking

**DOI:** 10.3390/s21062236

**Published:** 2021-03-23

**Authors:** Sichun Du, Qing Deng

**Affiliations:** College of Computer Science and Electronic Engineering, Hunan University, Changsha 410082, China; dengqing0832@hnu.edu.cn

**Keywords:** unscented particle filter, divide-and-conquer sampling, target tracking, accuracy, algorithm redundancy

## Abstract

Unscented particle filter (UPF) struggles to completely cover the target state space when handling the maneuvering target tracing problem, and the tracking performance can be affected by the low sample diversity and algorithm redundancy. In order to solve this problem, the method of divide-and-conquer sampling is applied to the UPF tracking algorithm. By decomposing the state space, the descending dimension processing of the target maneuver is realized. When dealing with the maneuvering target, particles are sampled separately in each subspace, which directly prevents particles from degeneracy. Experiments and a comparative analysis were carried out to comprehensively analyze the performance of the divide-and-conquer sampling unscented particle filter (DCS-UPF). The simulation result demonstrates that the proposed algorithm can improve the diversity of particles and obtain higher tracking accuracy in less time than the particle swarm algorithm and intelligent adaptive filtering algorithm. This algorithm can be used in complex maneuvering conditions.

## 1. Introduction

The problem of nonlinear filtering is a hot topic in signal processing and control theory [1,2]. It has wide applications in many fields, such as radar tracking [3,4,5], signal processing [6,7,8], mobile robot [9,10], and navigation [11,12]. The optimal nonlinear filter equation was developed in the mid-1960s, but the problem of the integration involved is still difficult to handle. In a linear Gaussian system, the Kalman filter (KF) [13] is the best estimate. However, in actual application, most systems are nonlinear/non-Gaussian. For such systems, KF will become invalid. In order to solve this problem, a method of approaching the nonlinear state-space model with the Kalman filter is proposed, namely, the extended Kalman filter (EKF), which uses Taylor series expansion instead of state transition equation and measurement equation [14,15,16], but, for strong non-linear systems, this method will bring large truncation errors [17,18]. Meanwhile, a complicated computation process of dealing with the Jacobian matrix is also related to the EKF. Then, the unscented Kalman filter (UKF) is proposed, which uses several sigma points to recursively calculate the mean and covariance [19]. The problems in the EKF have been solved, but the UKF still can only use the Gaussian distribution to approach the true posterior distribution [20,21]. In the late 1990s, based on the sequential importance sampling (SIS) [22,23], Gordon proposed a particle filter (PF) algorithm [24,25] by combining the resampling technique with Monte Carlo importance sampling. This algorithm is an optimal regression algorithm, combining Monte Carlo thought [26] and recursive Bayesian filtering [27], and it has a good estimation effect when dealing with nonlinear/non-Gaussian systems [28,29,30]. However, particle degradation and particle shortage occur during particle sampling, which seriously affects the accuracy of the PF.

In order to solve the above-mentioned problems mentioned, the UKF and PF are merged and the UPF method is introduced to implement state estimation. However, when the dynamic system comes across the interferences of abnormal observation and serious model noise, degradation of the particle will still occur [31,32]. Most studies are devoted to improving particle resampling steps to solve this problem. The authors of [33,34,35] took the concept of adaptive robust filtering into the UPF, improving the degeneracy of particles. Then, Wei et al. [36] proposed a new filter, combining adaptive filtering and square-root filtering This not only has the advantages of the adaptive filtering and square-root filtering, it also has a higher tracking accuracy. Then, ref. [37] proposed a UPF which applies particle swarm optimization (PSO) to UKF, which further improved the filter performance. Liu et al. [38] proposed an improved UPF based on a genetic algorithm (GA-UPF). The GA algorithm is used to optimize the particles, which eliminates the blind optimization of particles in the re-sampling process and solves the problem of particle impoverishment. Ramazan Havangi [39] developed an intelligent adaptive unscented particle filter (IAUPF), which uses an adaptive UKF filter to generate the proposal distribution and uses the genetic operators to increase the diversity of particles. When the noise statistics are unknown, the IAUPF has good performance. Sample impoverishment was improved in the mentioned algorithm. However, when dealing with the maneuvering target tracking, due to the inconsistency of maneuvering modes and intensities in different target directions, the state space will have sparse particle distribution in some regions, which is difficult to cover uniformly. With the increase in the complexity of the target motion model, the performance of the algorithm decreases more obviously. It is often necessary to increase the number of particles to ensure that the coverage and the operation time are longer, which does not guarantee real-time tracking and good tracking accuracy.

Aiming to solve the above problems, the divide-and-conquer algorithm is introduced to UPF in this paper. Applying optimization algorithms to UPF can also solve the problems mentioned above. However, from the perspective of the algorithm, the divide-and-conquer algorithm has more advantages than the optimization algorithm [40,41,42]. The particle swarm algorithm in the swarm intelligence algorithm is applied to find the optimal investment allocation of the stocks [40]. Based on this method, we can obtain a more accurate estimation, but this algorithm performs a global optimization, which leads to a long running time. The divide-and-conquer algorithm divides the problem into many sub-problems, which not only guarantees the accuracy of the information, but also reduces the running time. The meta-heuristic algorithm is used to optimize the control parameters of the given chaotic systems [41]. The algorithm has been improved in terms of running time, but it is easy to prematurely fall into the local optimum during the optimization process. The acquisition of the optimal solution of the divide-and-conquer algorithm is merging the optimal solutions of each sub-problem, and this will not fall into the local optimal situation. In [42], an improved heuristic algorithm–tabu search (TS) algorithm is used to deal with disturbances and variations in the nonlinear systems. This TS algorithm improves the shortcomings in [41], but it has a strong dependence on the initial solution, and the iterative process is serial to ensure that the algorithm runs longer, which is a common problem with most optimization algorithms. In the divide-and-conquer algorithm, the sub-problems at the same level can be processed in parallel, which reduces the running time.Through the above comparison, we can find that the divide-and-conquer algorithm has a greater advantage in terms of accuracy and computational performance, and can better solve the problem of sparse particle distribution in certain regions in the UPF.

This paper proposes a target tracking algorithm based on DCS-UPF. This algorithm solves the problem of sparse particle distribution in state space by decomposing state space, thus reducing the impact of particle degradation and particle shortage on the tracking performance. At the same time, it reduces the dimensionality of the motion space, which simplifies the algorithm-processing process and decreases the running time, thereby ensuring real-time tracking and good tracking accuracy. Experiments and a comparative analysis were carried out to comprehensively analyze the performance of DCS-UPF.

The structure of this paper is organized as follows. Section 2 defines the tracking model which is applied in this paper. Section 3 gives an overview of the fundamentals of the UPF. Section 4 introduces the algorithm of DCS-UPF. Section 5 presents the results of the simulation, which are used to demonstrate the effectiveness of DCS-UPF. In the end, Section 6 provides a conclusion.

## 2. General Tracking Models

Considering the model of target state as follows

(1) $$X_{k}=F_{k}X_{k-1}+w_{k}$$

where $X_{k}$ is the state vector at time *k*, $F_{k}$ is the system state transition function, and $w_{k}$ is the input process noises, which are entirely unrelated to the past and current states. Meanwhile, supposing $w_{k}$ is already known, which means that the probability density function is giving at first. It is obvious that Equation (1) provides the process of a first Markov. The target state includes position, velocity, acceleration, etc. In this paper, the vector $X_{k}$ is defined as: $X_{k}=\left[x_{k},{\bar{x}}_{k},y_{k},{\bar{y}}_{k}\right]$.

When the state vector $X_{k}$ is known, the measurement can compute via the measurement model. The measurement model is defined as follows

(2) $$Z_{k}=H_{k}X_{k}+v_{k}$$

where $Z_{k}$ is the measurement vector at time *k*, $H_{k}$ is the measurement function, and $v_{k}$ is another measurement noise vector which is also entirely unrelated to the past and current states. Meanwhile, $v_{k}$ is already known, which means that the probability density function is giving at first. In the motion space, the vector $Z_{k}$ is defined as: $Z_{k}={\left[x_{k},y_{k}\right]}^{T}$.

In practical applications, the observation data are based on the polar or spherical coordinates obtained by the radar sensor, including radial distance *r*, azimuth angle *b*, and pitch angle *e*. When the system observations are known $Z={\left[r,b\right]}^{T}$, obtaining the observations in Cartesian coordinates

(3) $$Z_{c}=\left[\begin{array}{c} x\\ y \end{array}\right]=\Phi \left(Z\right)=\left[\begin{array}{c} r\cos b\\ r\sin b \end{array}\right]$$

Supposing there is a coordinate transformation between two coordinates, $\Phi =h^{-1}$ and $h={\left[h_{r},h_{b}\right]}^{⊤}$. The real observation data in the Cartesian coordinate system after conversion can be expressed as

(4) $$\left\{\begin{array}{c} h_{x}=x+v_{x}\\ h_{y}=y+v_{y} \end{array}\right.$$

where $v_{x}$ and $v_{y}$ are the measurements of noise in the radial distance and azimuth angle.

In the following section, suppose that these rules are true:

(i) The system state transition function and the measurement function are practicable;
(ii) The states are related to a Markov process and the measurements which are independent correspond to the states;
(iii) The probability density functions of $w_{k}$ and $v_{k}$ are already known.

## 3. Fundamental of Unscented Particle Filter

UKF obtains a set of sigma sampling particles via the method of unscented transformation (UT), which can be taken as the posterior probability distribution. It is also a recursive Bayesian estimation method. Under the framework of PF, the basic idea of the UPF algorithm is to use UKF to generate the proposed distribution to guide PF sampling, and then to use the PF algorithm to predict the state and obtain the state estimation. The iterative calculation of the UPF makes full use of the measurement information at the latest time in every step, and the sampled particles can better approach the true value of the poster distribution. Meanwhile, UPF inherits the flexibility of the PF, which can change the estimation accuracy by adjusting the number of particles. The following shows the UPF algorithm:

Step 1: Initialization: when k=0, based on the initial state variable $X_{0}$, the particle set $\left\{X_{0,i}^{1}i=1,2,\cdots ,N\right\}$ are generated from the initial distribution $P\left(X_{0}\right)$. The initial weight of each particle is 1/N, and *N* represents the total number of particles;

Step 2: When k>0, the particle set generated from step (k−1) is updated to obtain the particle set of step *k* via formulas (1) and (2), and then the posterior probability distribution of step *k* is approximately described by the updated particle set. This process includes: using UKF to generate importance density function, importance sampling, calculating importance weight and normalizing processing, judging whether resampling is needed, outputting estimation results, and so on. These processes are described in detail as follows:

- Generate the importance density function for each particle by UKF;
- Constructe the sigma sampling point set and weight value for each particle, and the UT transformation is realized by the symmetric sampling strategy
  (5) $$\left.\chi_{k-1}^{i}=\lfloor x_{k-1}^{i}x_{k-1}^{i}+\sqrt{\left(L+\lambda \right)p_{k-1}^{i}}x_{k-1}^{i}-\sqrt{\left(L+\lambda \right)p_{k-1}^{i}}\right\rfloor$$
  (6) $$W_{j,m}=\left\{\begin{array}{cc} \frac{\lambda }{\lambda +L} & j=0\\ \frac{1}{2(\lambda +L)} & j=1,2,\ldots 2L \end{array}\right.$$
  (7) $$W_{j,m}=\left\{\begin{array}{cc} \frac{\lambda }{\lambda +L}+\left(1+\beta -\alpha^{2}\right) & j=0\\ \frac{1}{2(\lambda +L)} & j=1,2,\ldots 2L \end{array}\right.$$
  where $\chi_{k-1}^{i}$ stands for the *i*th sigma point, $W_{j,m}$ stands for the weight of the *j*th sigma point, *L* is the dimension of state variable, α is a proportional correction factor, ranging from $10^{-4}$ to 1. For the parameters β, the optimal number is 2 under the Gaussian distribution, κ is a secondary sampling factor which usually uses 0 or (3−L). $\lambda =\alpha^{2}\left(L+\kappa \right)-L$ is the fine-tuning parameter, and $P_{k-1}^{i}$ is the state covariance matrix for each particle;
- Calculate predicted mean and covariance of each particle via a one-step prediction of sigma sampling points
  (8) $$x_{k|k-1}^{i}=f\left(\chi_{k-1}^{i}\right)+q_{k-1}$$
  (9) $${\bar{x}}_{k|k-1}^{i}=\sum_{j=0}^{2L}W_{j,m}x_{k|k-1,j}^{i}$$
  (10) $${\bar{P}}_{k|k-1}^{i}=\sum_{j=0}^{2L}\left[W_{j,c}\left({\bar{x}}_{k|k-1}^{i}-x_{k|k-1,j}^{i}\right){\left({\bar{x}}_{k|k-1}^{i}-x_{k|k-1,j}^{i}\right)}^{T}\right]+Q_{k|k-1}$$
- Reconstructe the sigma point set based on the predicted value and predicted covariance mentioned in Equations (9) and (10)
  (11) $$\gamma_{k-1}^{i}=\left\lfloor {\bar{x}}_{k-1}^{i}{\bar{x}}_{k-1}^{i}+\sqrt{\left(L+\lambda \right){\bar{p}}_{k-1}^{i}}{\bar{x}}_{k-1}^{i}-\sqrt{\left(L+\lambda \right){\bar{p}}_{k-1}^{i}}\right\rfloor$$
  (12) $$z_{k|k-1}^{i}=h\left(\gamma_{k|k-1}^{i}\right)+r_{k}$$
  (13) $${\bar{z}}_{k|k-1}^{i}=\sum_{j=0}^{2L}W_{j,m}z_{k|k-1,j}^{i}$$
- Calculate the self-covariance and mutual-covariance
  (14) $$P_{zz}^{i}=\sum_{j=0}^{2L}\left[W_{j,c}\left({\bar{z}}_{k|k-1}^{i}-z_{k|k-1,j}^{i}\right){\left({\bar{z}}_{k|k-1}^{i}-z_{k|k-1,j}^{i}\right)}^{T}\right]+R_{k}$$
  (15) $$P_{xz}^{i}=\sum_{j=0}^{2L}\left[W_{j.c}\left({\bar{x}}_{k|k-1}^{i}-x_{k|k-1,j}^{i}\right){\left({\bar{z}}_{k|k-1}^{i}-z_{k|k-1,j}^{i}\right)}^{T}\right]$$
- Calculate the Kalman gain, and the particles are updated with the latest measurement to produce the importance density function
  (16) $$K^{i}=P_{xz}^{i}{\left(P_{zz}^{i}\right)}^{-1}$$
  (17) $${\bar{x}}_{k}^{i}={\bar{x}}_{k|k-1}^{i}+K^{i}\left(z_{k}-{\bar{z}}_{k|k-1}^{i}\right)$$
  (18) $${\bar{P}}_{k}^{i}={\bar{P}}_{k|k-1}^{i}-K^{i}P_{zz}^{i}{\left(K^{i}\right)}^{T}$$
- Sample particles from the importance density function
  (19) $${\bar{x}}_{k}^{i}∼q\left(x_{k}^{i}|x_{0:k-1}^{i},z_{1:k}\right)=N\left({\bar{x}}_{k}^{i},{\bar{P}}_{k}^{i}\right)$$
- Calculate the weight of each particle in Formula (19) and normalize them
  (20) $$\omega_{k}^{i}=\omega_{k-1}^{i}\frac{p\left(z_{k}|{\bar{x}}_{k}^{i}\right)p\left({\bar{x}}_{k}^{i}|{\bar{x}}_{k-1}^{i}\right)}{q\left(x_{k}^{i}|x_{0:k-1}^{i},z_{1:k}\right)}$$
  (21) $${\bar{\omega }}_{k}^{i}=\frac{\omega_{\hat{k}}^{i}}{\sum_{i=1}^{N}\omega_{k}^{i}}$$
- Compare $N_{\mathrm{eff}}$ with $N_{\mathrm{th}}$ and set $N_{\mathrm{th}}$ to N/3. If $N_{\mathrm{eff}}\leq N_{\mathrm{th}}$, perform re-sampling, otherwise skip this step and continue. Resampling includes random resampling, polynomial resampling, system resampling, residual resampling, etc. This paper uses random resampling to realize resampling.
  (22) $$N_{\mathrm{eff}\;}=\frac{1}{\sum_{i=1}^{N}{\left({\bar{\omega }}_{k}^{i}\right)}^{2}}\leq N_{\mathrm{th}\;}$$
- Output estimation results:
  (23) $${\bar{x}}_{k}=\sum_{i=1}^{N}{\bar{\omega }}_{k}^{i}{\bar{x}}_{k}^{i}$$
  (24) $${\bar{P}}_{K}=\sum_{i=1}^{N}{\bar{\omega }}_{k}^{i}\left({\bar{x}}_{k}-{\bar{x}}_{k}^{i}\right){\left({\bar{x}}_{k}-{\bar{x}}_{k}^{i}\right)}^{T}$$
- Go to step 2 for the next iteration.

## 4. The Algorithm of Divide-and-Conquer Sampling Unscented Particle Filter

### 4.1. The Method of Divide-and-Conquer Sampling

For the PF algorithm, the higher the dimension of state space, the higher the number of particles required, thus ensuring that the spatial coverage of particles is wide enough. With the increase in state dimension, the complexity of the algorithm increases exponentially. These characters also hold in the UPF algorithm. The basic idea of the divide-and-conquer algorithm is to decompose a problem of scale *N* into *K* smaller-scale sub-problems, which are independent of each other and have the same properties as the original problem. When the solution to the sub-problem is found, you can obtain the solution to the original problem.

**Figure 1:**
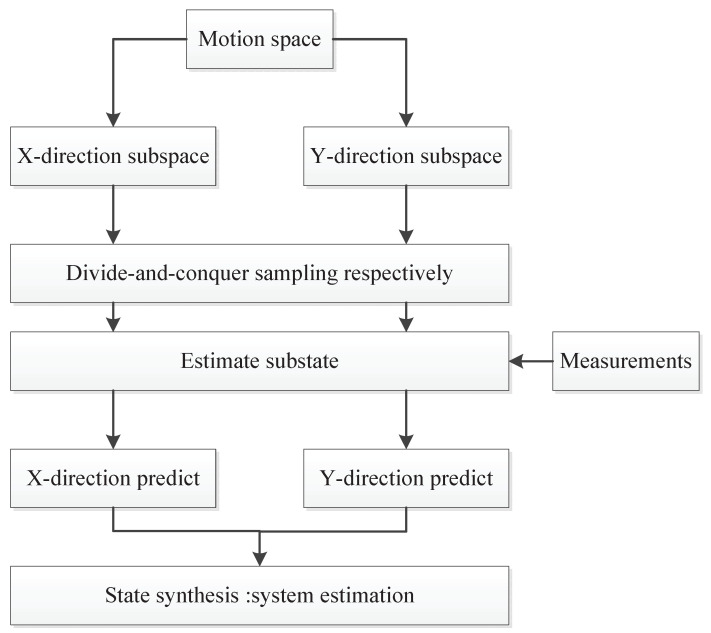
The flowchart of spatial divide-and-conquer sampling method.

It is noted that, in the coordinate system, the motion states of the target in each direction of motion space are independent from each other and not affected by the motion in other directions. In the other words, the motion space is orthometric. According to the idea of motion decomposition and synthesis, the whole motion state of the target can be expressed as the superposition of motion in each direction. The flowchart of the spatial divide-and-conquer sampling method is shown in Figure 1. The motion space is divided into two directions: X and Y. The state is estimated by sampling in the subspace. Then, the state of each subspace is predicted with the measurement information. Finally, the subspace information is merged.

The idea of the divide-and-conquer method is introduced to segment motion space. Assuming that the particles are sampled in the one-dimensional subspace and the random samples $N_{x}$, $N_{y}$ are sampled in each direction, then the total number of particles is ($N_{x}+N_{y}$), and the total particle diversity performance reaches ($N_{x}*N_{y}$). When sampling directly in the state space, there will be only ($N_{x}+N_{y}$) species. It is obvious that the spatial coverage of the former sampling particles is wider, and the former can reduce the particle degradation phenomenon to some extent and improve the prediction accuracy.

The complexity of the algorithm is analyzed below. According to Section 2 of this paper, the dimension of the state vector is 4 and the dimension of the observation vector is 2. Let the total number of particle sampling be N, the state one-step prediction time in the filtering algorithm is $T_{f}$, and the measurement one-step prediction time is $T_{h}$. When the state space is decomposed into two independent subspaces where the same number of samples are taken, the prediction time of state one-step is reduced to $T_{f}$/4, and the prediction time of measurement one-step is reduced to $T_{h}$/2. For the same model, the complexity of traditional sampling filtering method is N*($T_{f}$+ $T_{h}$) and that of divide-and-conquer sampling filtering is N*($T_{f}$+ 2$T_{h}$)/4. The complexity of the two methods is the same order, but the operation time of the divide-and-conquer sampling method is smaller than that of the UPF.

### 4.2. Unscented Particle Filter Tracking Algorithm Based on Divide-and-Conquer Sampling

**Figure 2:**
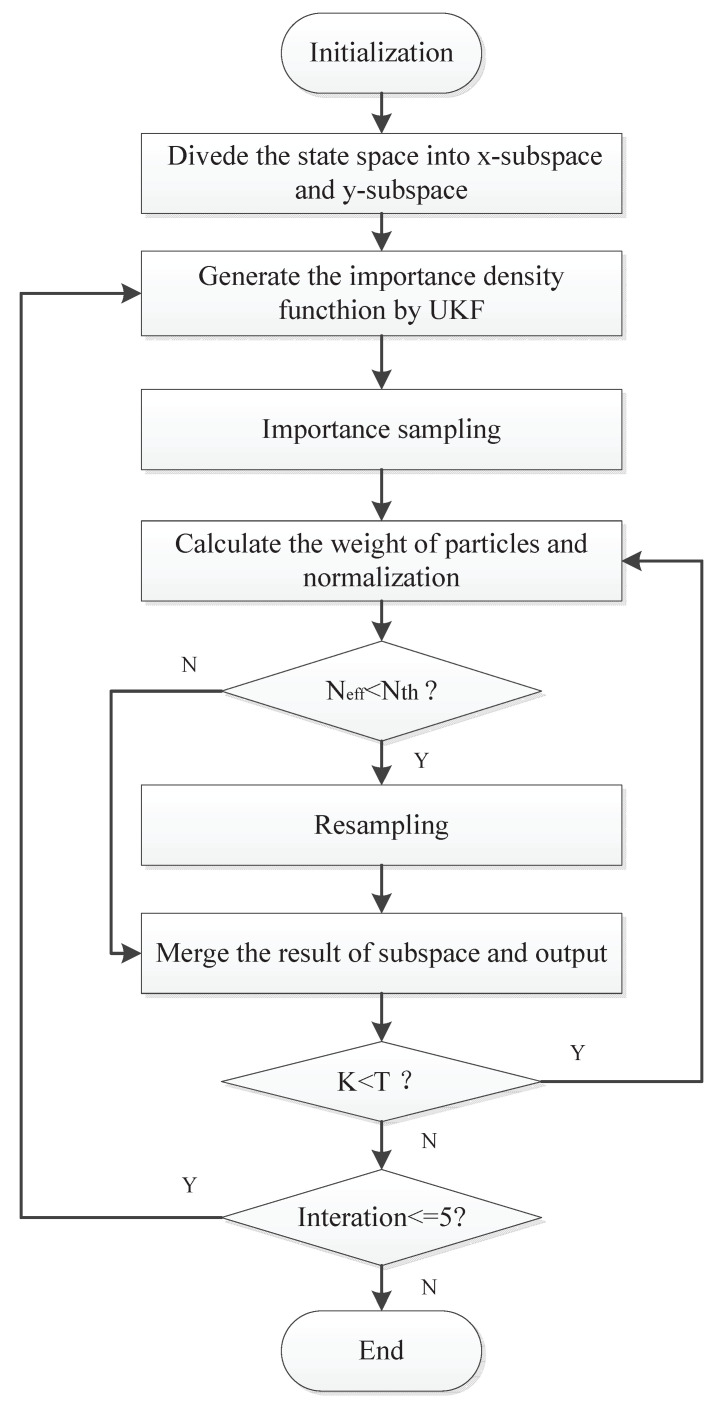
The flowchart of the divide-and-conquer sampling unscented particle filter.

The basic idea of the maneuvering target-tracking algorithm based on DSC-UPF is to decompose the motion space into two independent, one-dimensional state subspaces according to the Cartesian coordinates system. The final output state is obtained using an UPF algorithm according to the optimal sampling strategy. The step of a UPF tracking algorithm based on divide-and-conquer sampling is described as follows, and the flowchart is shown in Figure 2.

(1) Initialization: When k=0, the subspace is decomposed according to the state space independence, and the one-dimensional subspace particle set ${\left\{X_{x0}^{i},w_{x0}^{j}\right\}}_{i=1}^{N_{z}}$,
  ${\left\{Y_{y0}^{i},w_{y0}^{i}\right\}}_{i=1}^{N}$ are generated by the prior probability $P\left(x_{0}\right)$;
(2) Predict: When k=1,2,…,T, the UPF algorithm is run for each particle set, and the state estimation in each subspace $\left({\hat{X}}_{xk},{\hat{Y}}_{yk}\right)$ is obtained;
(3) Output Synthesis state: According to each sub-state estimate, obtaining the total state estimation.

## 5. Simulation Results and Analysis

In order to verify the effectiveness of the algorithm, simulation experiments are carried out. In the simulation experiment, the algorithm is compared with the standard PF and UPF at first, and then compared with the unscented particle filter based on the particle swarm optimization algorithm (PSO-UPF) and the unscented particle filter based on an intelligent adaptive algorithm (IA-UPF). The goal of the target tracking is to obtain the moving target’s position via measurements. The state of the target at time *k* consists of the position and velocity. The state vector and measurement vector are defined as

(25) $$X_{k}=\left[x_{k},{\bar{x}}_{k},y_{k},{\bar{y}}_{k}\right]$$

(26) $$Z_{k}=H_{k}X_{k}+v_{k}$$

Setting the sensor’s sampling interval is 1 s, and the total time of sampling is 60 s. Meanwhile, both process noise and measurement noise obey Gaussian distribution (N(0,1)). The initial state of the target is $X_{0}=\left[1m,1m,1m/s,1m/s\right]$. The motion model is the variable speed motion model

(27) $$F=\left[\begin{array}{cccc} 1 & 0 & at & 0\\ 0 & 1 & 0 & bt\\ 0 & 0 & a & 0\\ 0 & 0 & 0 & b \end{array}\right]$$

**Figure 3:**
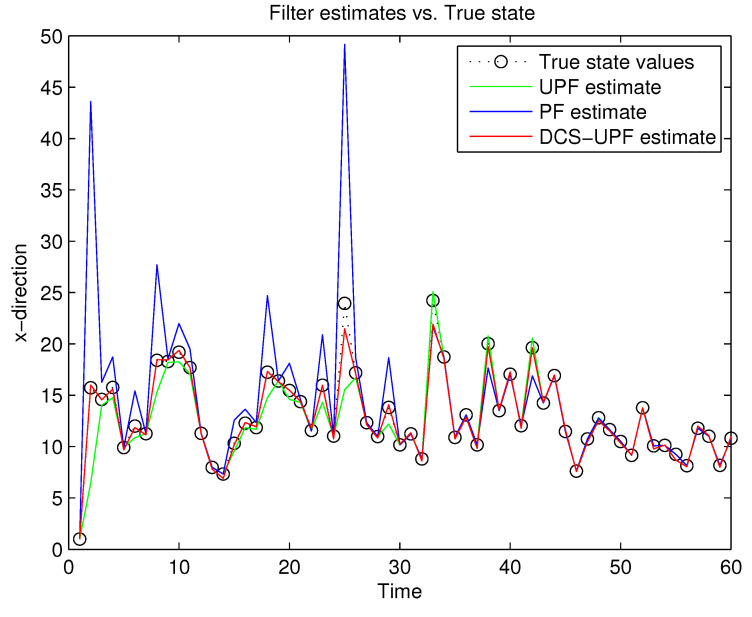
Target trajectory in X-direction.

**Figure 4:**
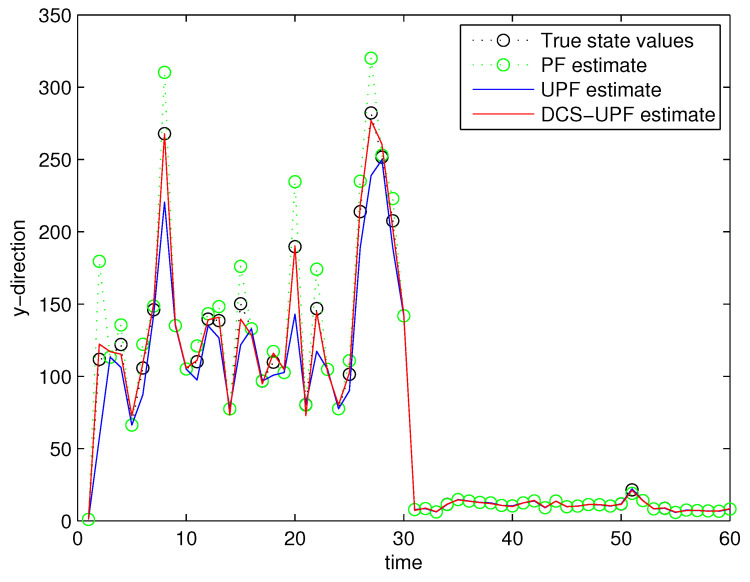
Target trajectory in Y-direction.

This simulation study is performed in MATLAB 2014b coding environment on a desktop computer with Intel Core i5-4700, 3.6 GHz, 64-bit Windows 7 operating system. The simulation starts at time k=0, when the first measurement of the target is obtained. This may be viewed as the time when the target is first detected. Figure 3 and Figure 4 demonstrate the performance using PF, UPF, and DCS-UPF to track the target in each subspace. In Figure 3 (Figure 4), the red curve represents the tracking curve of DSC-UPF. The green (blue) curve represents the tracking curve of the UPF. The blue (green) curve represents the tracking curve of the PF. The true state value is shown by the black curve. DCS-UPF has produced the best tracking performance. Figure 3 describes the tracking performance of three filters in the X-direction. For most states, it is clear that DCS-UPF tracks the target more accurately than PF and UPF. Meanwhile, the curve of DCS-UPF almost overlaps with the true state values, which means that DCS-UPF can be used to represent the true state value in some cases. The tracking performance of three filters in the Y direction is shown in Figure 4. It can be seen that PF and UPF estimate the state of position incorrectly in many cases. However, DCS-UPF provides much more accurate estimation results. The reason for this phenomenon is that, firstly, particle degradation seriously affects the tracking accuracy of PF. This is because PF takes the importance density function (recommended distribution) as equal to the prior distribution, the latest measurement information is not considered, and the sampled particles cannot effectively approach the true value of the posterior probability distribution. In addition, the full prior knowledge of noise statistics is not provided. Then, UPF weakens particle degradation by generating important functions and resampling using UT. However, UPF struggles to completely cover the target state space when handling the maneuvering target tracking problem, and the tracking performance can be affected by the low sample diversity. The divide-and-conquer sampling algorithm is introduced into the UPF to solve the above problems. It successfully solves the problem of sparse particle coverage by sampling in subspace alone. Simulation results show that this method is effective in improving tracking performance. Looking at a comprehensive measurement of these two pictures, DSC-UPF has better tracking performance. The simulation results of Figure 3 and Figure 4 verified that the divide-and-conquer sampling algorithm can improve the poor tracking performance caused by the uneven distribution of particles in each subspace.

**Figure 5:**
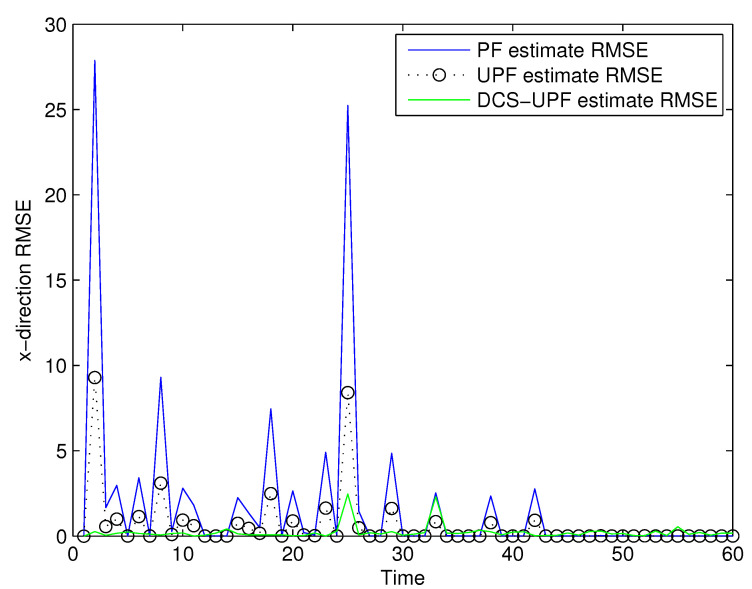
The tracking errors of three filters in X-direction.

**Figure 6:**
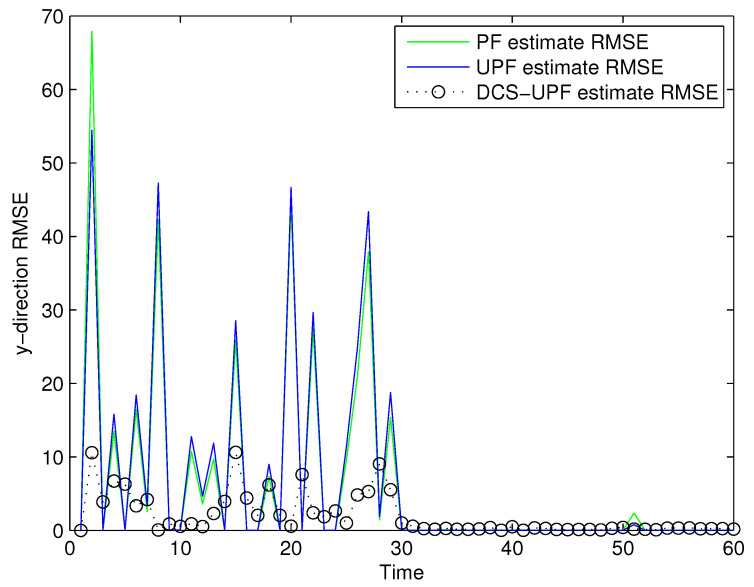
The tracking errors of three filters in Y-direction.

The tracking errors of the three filters are shown in Figure 5 and Figure 6. In Figure 5, the black curve represents the error of the DSC-UPF. The green curve represents the error of the UPF. The blue curve represents the error of the PF. The tracking errors of three filters in the X-direction are described in Figure 5. It can be observed that the tracking error of DCS-UPF is smaller than that of PF and UPF. Calculating the error variance, DCS-UPF is also smaller than the others. The tracking error of three filters in the Y direction is shown in Figure 6. The conclusion is the same as that in the X direction. In Figure 5 and Figure 6, the performance of DSC-UPF is measured from the perspective of tracking error, and the target tracking accuracy of DSC-UPF is higher. The results of the simulation show that the divide-and-conquer sampling algorithm can improve the tracking performance of UPF.

**Figure 7:**
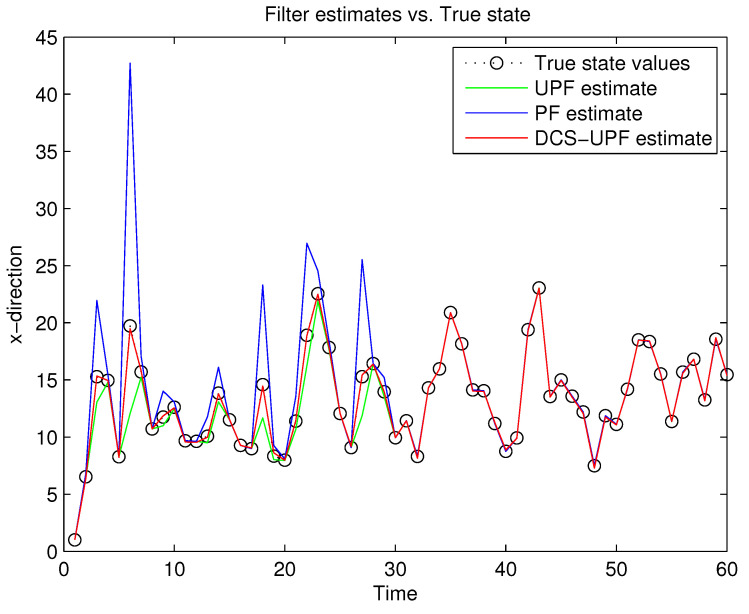
Target estimation in X-direction by changing R and Q.

**Figure 8:**
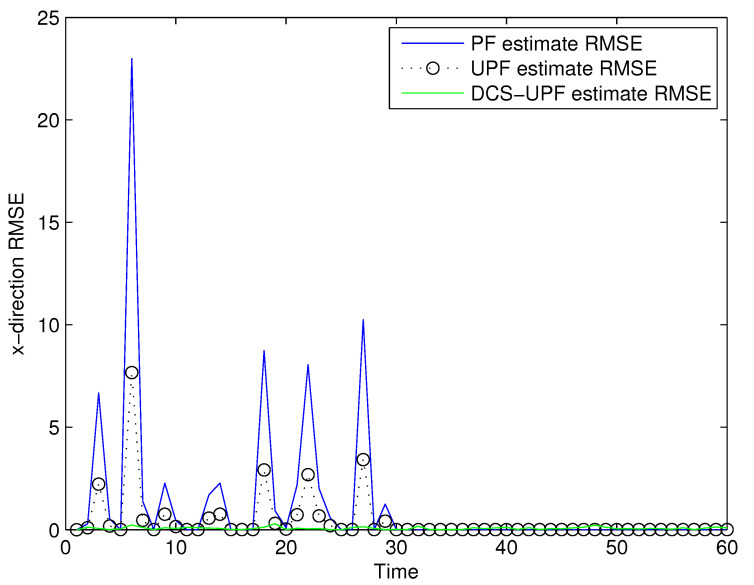
The tracking errors of three methods in X-direction by changing R and Q.

**Figure 9:**
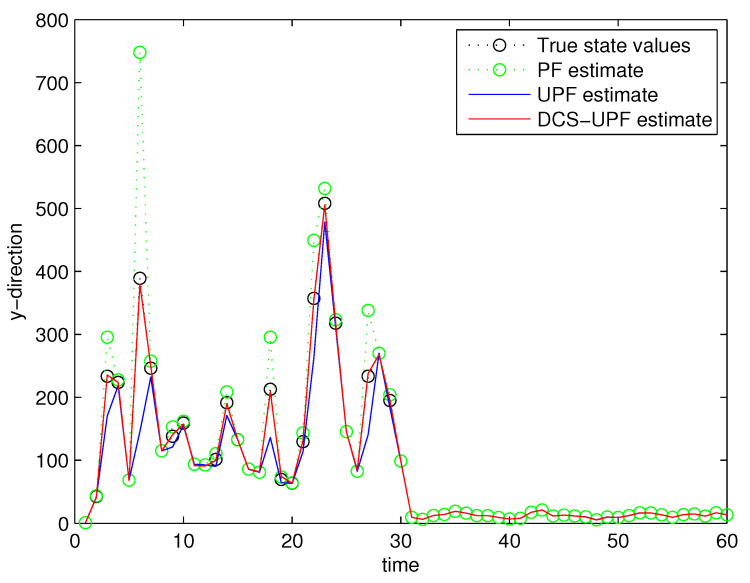
Target estimation in Y-direction by changing R and Q.

**Figure 10:**
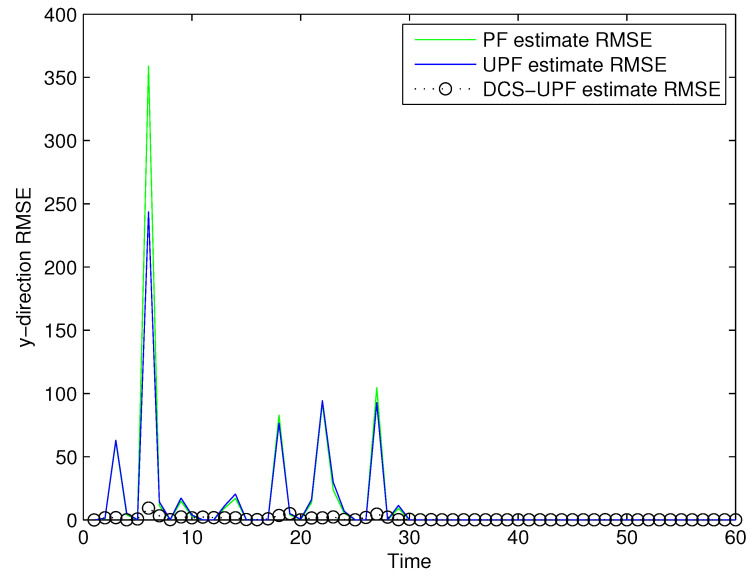
The tracking errors of three methods in Y-direction by changing R and Q.

The effect of Q and R on tracking performance is studied by changing the values of Q and R. The simulation results are shown in Figure 7, Figure 8, Figure 9 and Figure 10. As shown in the picture, changing the value of Q and R can obtain a better filter performance. Comparing Figure 3 (Figure 4) with Figure 7 (Figure 9), it can be seen that the performance of the filter in Figure 7 (Figure 9) has been improved. On the other hand, the tracking errors of filters shown in Figure 8 and Figure 10 indicate an improvement in the tracking performance. The simulation results show that the tracking accuracy of filters can be improved by changing the values of Q and R. Therefore, we can find suitable Q and R by constantly trying the corresponding values in the simulation process. This process can make the filter more accurate.

**Figure 11:**
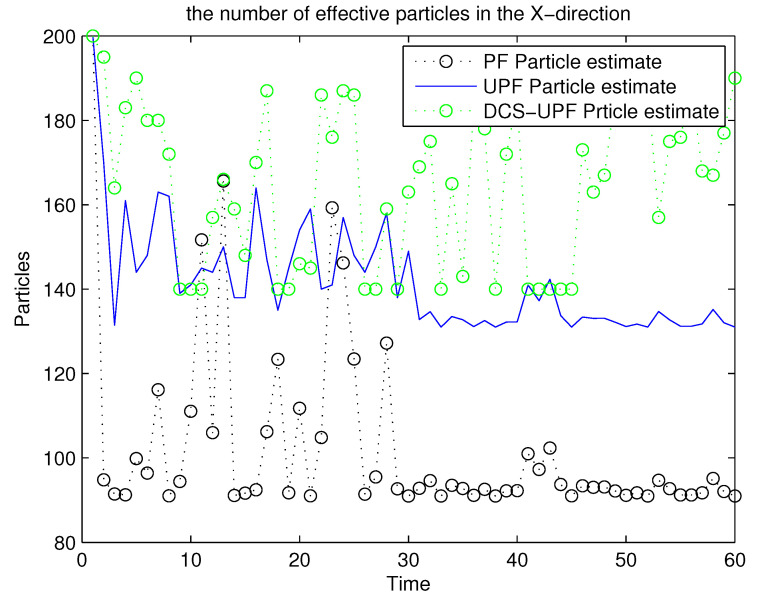
The effective particles numbers of three filters in X-direction.

**Figure 12:**
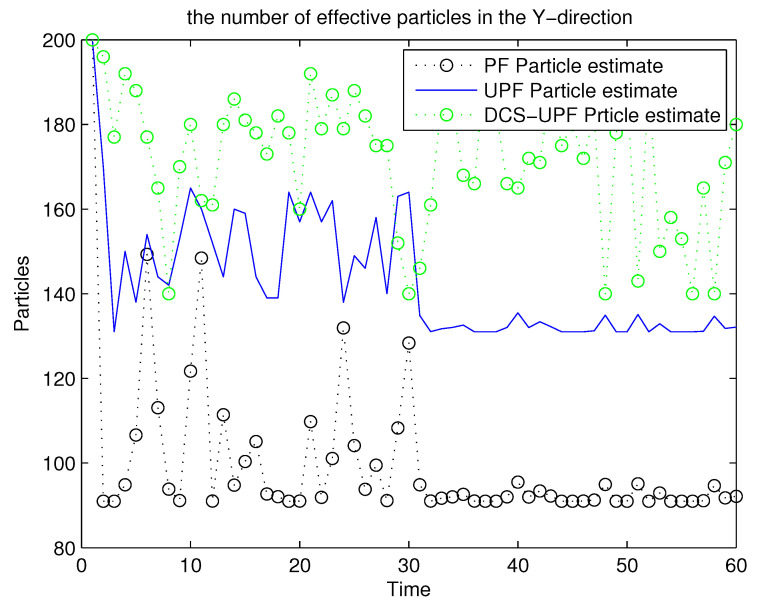
The effective particles numbers of three filters in Y-direction.

For the UPF algorithm, the accuracy of the trajectory tracking depends on the number of particles. Within a certain range, they are directly proportional. Figure 11 and Figure 12 demonstrate the number of effective particles in PF, UPF, and DCS-UPF. The number of effective particles in PF, UPF and DSC-UPF are represented by the blue curve, black curve and green curve, respectively. We can also find that the DSC-UPF has more effective particle numbers. The reason for this phenomenon is that particles are sampled separately in each subspace, so the number of particles distributed in each subspace becomes increasingly uniform. Meanwhile, it solves the problem of sparse particle distribution in the subspace. Therefore, DSC-UPF has much higher accuracy than both PF and UPF.

**Figure 13:**
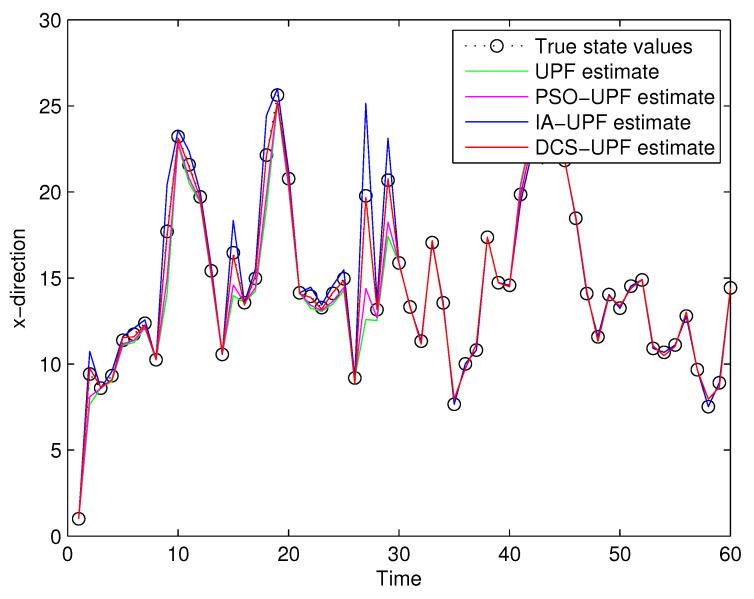
The target trajectory of UPF, PSO-UPF, IA-UPF, DSC-UPF in X-direction.

**Figure 14:**
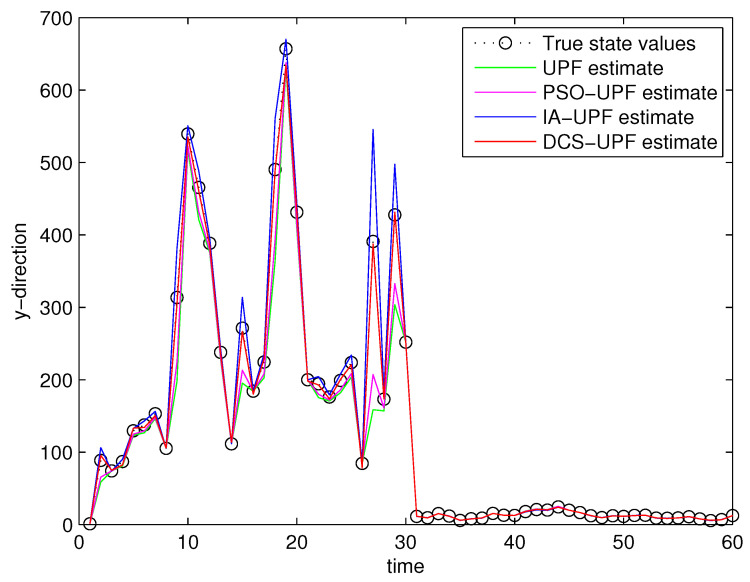
The target trajectory of UPF, PSO-UPF, IA-UPF, DSC-UPF in Y-direction.

For comparison analysis, trials based on the above experimental design were conducted by using UPF, PSO-UPF, IA-UPF, DSC-UPF, respectively. Figure 13 and Figure 14 show the simulation result. It can be seen that UPF has a poor tracking performance as the UKF uses only second-order moments, which may not be sufficient for some nonlinear systems. Moreover, the number of sigma points is small and may not represent complicated distributions. In addition, the resampling step leads to a loss of diversity among the particles, reducing the estimation accuracy. Although PSO-UPF enhances the tracking accuracy of UPF, the enhanced tracking accuracy is still limited. This is because particle swarm optimization (PSO) easily falls into the local optimum, which leads to low convergence accuracy and difficult convergence. Because IA-UPF reduces the loss of particle diversity caused mainly by the particle degradation in the resampling step and incorrect a priori knowledge of process and measurement noise. IA-UPF has much higher accuracy than both UPF and PSO-UPF. However, its filtering accuracy is significantly degraded when the distribution of particles in some areas is sparse. The emergence of DSC-UPF solves this problem, and the simulation results show that DSC-UPF has much higher accuracy than UPF, PSO-UPF and IA-UPF. Table 1 lists the average RMSE of UPF, PSO-UPF, IA-UPF and DSC-UPF. It can be seen that the average RMSE of DSC-UPF is minimal, which also illustrates that the tracking performance of DSC-UPF is more accurate.

**Figure 15:**
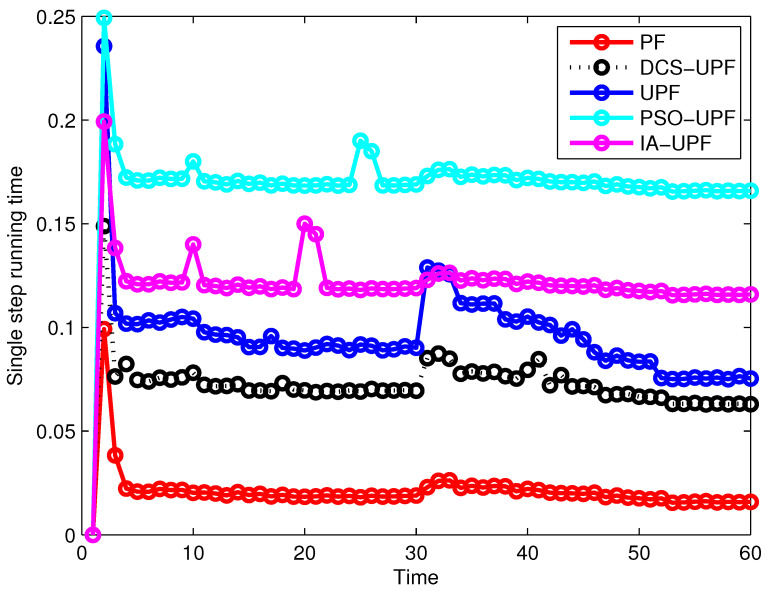
The one-step running time of PF, UPF, PSO-UPF, IA-UPF and DCS-UPF.

The one-step running times of PF, UPF, PSO-UPF, IA-UPF and DCS-UPF are shown in Figure 15. It can be seen that PF has the minimum one-step running time. This is because the simulation process of the PF is simpler and does not require UKF to generate the proposed distribution function. The PSO-UPF has the longest single-step running time, because PSO takes a lot of time to update the velocity and position of each particle in the process of UKF generating the proposed distribution. The single-step running time of IA-UPF is between PSO-UPF and UPF. The IAUPF uses an adaptive UKF to generate the proposal distribution and uses the genetic operators to increase the diversity of particles, so that the single-step running time of IA-UPF is higher than UPF. Moreover, the execution process of IA-UPF is relatively simple, and takes less time than PSO-UPF. The single-step running time of the proposed algorithm in this paper is only second to PF, because, with the decomposition of the motion space, the time of single-step iteration of the proposed algorithm on the basis of UPF is reduced.

Table 2 shows the computational performances of PF, UPF, PSO-UPF, IA-UPF and DSC-UPF. T*_f_* represents the state one-step prediction time in the filtering algorithm, T*_h_* represents the measurement one-step prediction time, and A<C<B. As shown in Table 2, the computational time of UPF, PSO-UPF, IA-UPF and DSC-UPF are notably larger than PF. This is because the computational processes of these four filters are more complex, involving the use of UKF to generate the proposed distribution, etc. Thus, they require more computational time and CPU utilization. The total running time and CPU utilization of the DSC-UPF are smaller than that of the UPF, PSO-UPF and IA-UPF. The reason for this appearance is that the divide-and-conquer algorithm divides the problem into many sub-problems, which not only guarantees the accuracy of the information but also reduces the running time. In addition, after the dimension reduction processing of the motion space, the running process of program is not as complicated as UPF, PSO-UPF and IA-UPF. In sum, the computational complexity of DSC-UPF is reduced. Combined with Figure 15, this conclusion can be confirmed further. As the complexity of the target state model increases, the algorithm performs better than UPF, PSO-UPF and IA-UPF.

## 6. Conclusions

This paper proposed a tracking algorithm based on DSC-UPF. By decomposing the independent state subspace, the reduction in dimension is realized, which solves the problem of sparse particle distribution and reduces the impact of particle degradation and particle shortage on the tracking performance. Compared with the standard UPF, PSO-UPF and IA-UPF, the simulation results verify that the algorithm proposed in this paper has significant advantages in tracking performance and computing performance. The reasons are as follows: firstly, it reduces the dimensionality of the motion space, which simplifies the algorithm processing process and decreases the running time, thereby ensuring real-time tracking and good tracking accuracy. Secondly, the particles are extracted from each subspace to estimate the state of this subspace, which solves the problem of particle shortage caused by uneven particle distribution in certain directions in the state space. In conclusion, the use of divide-and-conquer sampling algorithm in UPF greatly improves the tracking accuracy of the filter and reduces the complexity of the algorithm.

Future work is to consider the improvement in the resampling strategy, real-time performance, and robustness, as well as applying it to more fields.

## Figures and Tables

**Table 1:** The average RMSE of UPF, PSO-UPF, IA-UPF and DSC-UPF.

	UPF	PSO-UPF	IA-UPF	DSC-UPF
The average RMSE in X-direction	0.525	0.41	0.348	0.21
The average RMSE in Y-direction	5.63	4.32	4.14	1.67

**Table 2:** Computational performances of PF, UPF, PSO-UPF, IA-UPF and DSC-UPF.

	Time Complexity	One-Step Time/s	CPU Utilization	Total Time/s
PF	$O(Mn^{2})$	$T_{f}+T_{h}$	42%	1.54
UPF	$O(Mn^{2}+n^{3})$	$AT_{f}+T_{h}$	48%	6.25
PSO-UPF	$O(Mn^{3}+Nn^{3})$	$BT_{f}+T_{h}$	51%	10.1
IA-UPF	$O(Mn^{2}+Nn^{3})$	$CT_{f}+T_{h}$	49%	7.23
DSC-UPF	$O(Mn^{2}+n^{3})$	$(AT_{f}+2T_{h})/4$	47%	4.5

## Data Availability

No new data were created or analyzed in this study. Data sharing is not applicable to this article.
